# Invasive Urinary Tract Aspergillosis: A Rare Entity

**DOI:** 10.7759/cureus.80199

**Published:** 2025-03-07

**Authors:** Ahmed Amine Jaouahar, Zakaria Chahbi, Omar Maoujoud, Mohammed Asserraji, Nadir Zemraoui

**Affiliations:** 1 Nephrology and Hemodialysis, Avicenna Military Hospital, Marrakesh, MAR; 2 Infectious Disease, Avicenna Military Hospital, Marrakesh, MAR

**Keywords:** acute kidney injury, aspergillus fumigatus, hemodialysis, obstructive uropathy, urinary tract infections

## Abstract

We report a rare case of severe acute kidney injury (AKI) due to invasive urinary aspergillosis in a 59-year-old male. Our patient underwent extracorporeal shock wave lithotripsy (ESWL) for bilateral renal calculi followed by bilateral ureteral stent placement. Initially, urine bacteriological cultures were sterile, and renal function was normal. Days later, he developed rapidly progressive oligo-anuric AKI requiring hemodialysis. Despite stent replacement and empirical antibiotics, infection markers continued to rise, and the newly replaced stents became obstructed for the second time. Ureteroscopy-guided urine culture identified *Aspergillus fumigatus*. A few days after the initiation of voriconazole therapy, the patient demonstrated significant clinical and biological improvement, including urine clarification and normalization of renal function. This case highlights the need to consider fungal infections in persistent unexplained obstructive AKI and underscores the importance of early diagnosis and antifungal therapy.

## Introduction

Urinary aspergillosis, a rare and often elusive fungal infection, presents a significant diagnostic challenge in clinical practice. While *Aspergillus *species are commonly associated with pulmonary and systemic infections, urinary tract involvement remains an infrequent occurrence, typically seen in immunocompromised patients (HIV/AIDS, poorly controlled diabetes mellitus, prolonged corticosteroid therapy, intravenous drug use) or those with underlying urological conditions [1]. The difficulty in diagnosing urinary aspergillosis stems from its nonspecific symptoms, which often mimic more common urological disorders, and the lack of routine screening for fungal infections in the urinary tract. Consequently, early recognition and timely intervention are crucial, as delayed diagnosis can lead to complications, including renal damage or sepsis [2]. This article explores the clinical presentation, diagnostic hurdles, and treatment strategies for urinary aspergillosis, emphasizing its elusive nature and the importance of considering this rare pathogen in differential diagnoses. In a medical landscape where rapid detection is key, urinary aspergillosis often challenges clinicians to "catch it if they can," underscoring the need for heightened awareness and a systematic approach to diagnosis.

## Case presentation

We report a case of severe acute kidney injury (AKI) due to invasive urinary aspergillosis in a 59-year-old diabetic male.

The patient underwent extracorporeal shock wave lithotripsy (ESWL) for bilateral renal calculi with subsequent placement of bilateral ureteral stents. Initially, urine was sterile on cytobacteriological examination, creatinine serum levels were normal, and infection markers were negative. However, a few days later, our patient became anuric and a rapidly progressive AKI developed along with acute pulmonary edema, necessitating several sessions of hemodialysis (the patient underwent three daily consecutive sessions of intermittent hemodialysis, followed by one session every other day, for a total of 10 sessions). In view of his clinical deterioration and unexplained rise of infection markers, the ureteral stents were replaced, resulting in the immediate resumption of normal urine output (Table 1).

**Table 1:** Laboratory findings of the patient Biological severe acute kidney injury (AKI) and inflammatory syndrome

Laboratory parameters	Patient value	Normal range
White blood cells	15.2 x 10^9^	4.0-10 x 10^9^/L
Hemoglobin	11	12-16 g/dL
Platelets	321 x 10^9^	150-400 x 10^9^/L
C-reactive protein	210	< 0.3 mg/dL
Serum urea	41	2-10 mmol/L
Serum creatinine	829	60-119 µmol/L

Despite negative routine urine and blood cultures, the urine aspect remained purulent. Infection markers continued to rise regardless of the use of broad-spectrum antibiotic therapy (Ceftriaxone 2 g per day + Ciprofloxacin 200 mg twice a day), and within 24 hours, the new ureteral stents became obstructed for the second time (Table 2).

**Table 2:** Laboratory values of the patient days after initiating empiric antibiotherapy CRP and WBC counts continued to rise under broad-spectrum antibiotics

Laboratory parameters	Patient value	Normal range
White blood cells	18 x 10^9^	4.0-10 x 10^9^/L
Hemoglobin	10.2	12-16 g/dL
Platelets	380 x 10^9^	150-400 x 10^9^/L
C-reactive protein	289	< 0.3 mg/dL
Serum urea	38	2-10 mmol/L
Serum creatinine	620	60-119 µmol/L

**Figure 1:**
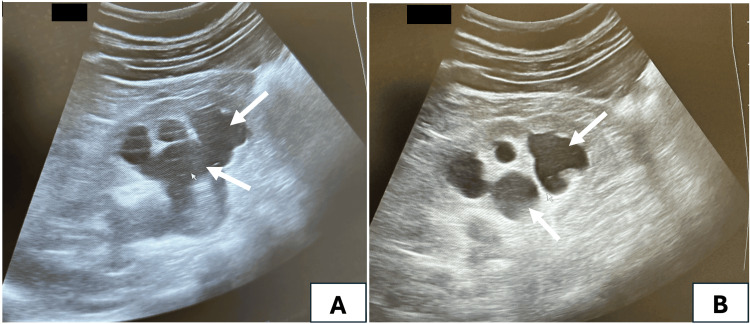
Renal ultrasound showing bilateral hydronephrosis (arrows) A: right kidney pyelocalyceal dilation; B: left kidney pyelocalyceal dilation

A renal ultrasound was performed, revealing two kidneys measuring 14 cm each in their longest axis, with preserved corticomedullary differentiation. Both pyelocalyceal cavities were found to be dilated without any evident obstruction cause (Figure 1).

**Figure 2:**
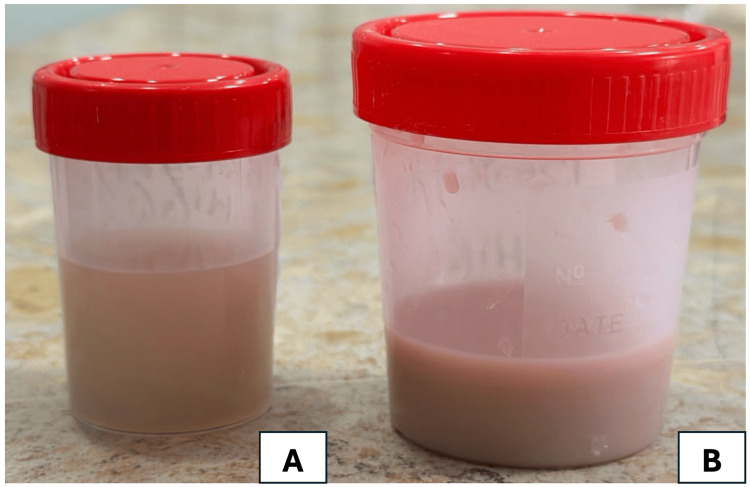
Aspect of pyelic urine collected via ureteroscopy A: left kidney sample; B: right kidney sample

A CT urography was not performed to avoid exacerbating renal impairment due to the nephrotoxicity of contrast agents. The obstructive component of AKI and the probable diagnosis of acute pyelonephritis associated with bilateral ureteral obstruction justified the indication for ureteral stents removal for the second time along with a bilateral ureteroscopy, which retrieved purulent urine with jelly-like texture (Figure 2).

**Figure 3:**
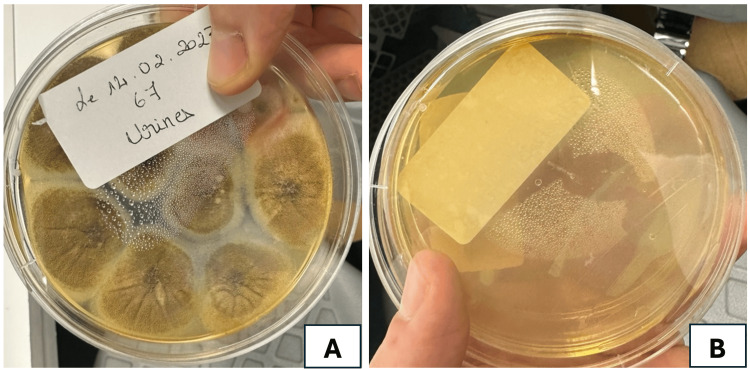
Mycological culture identifying colonies of Aspergillus fumigatus A: before treatment; B: sterile culture after only three days of IV voriconazole

**Figure 4:**
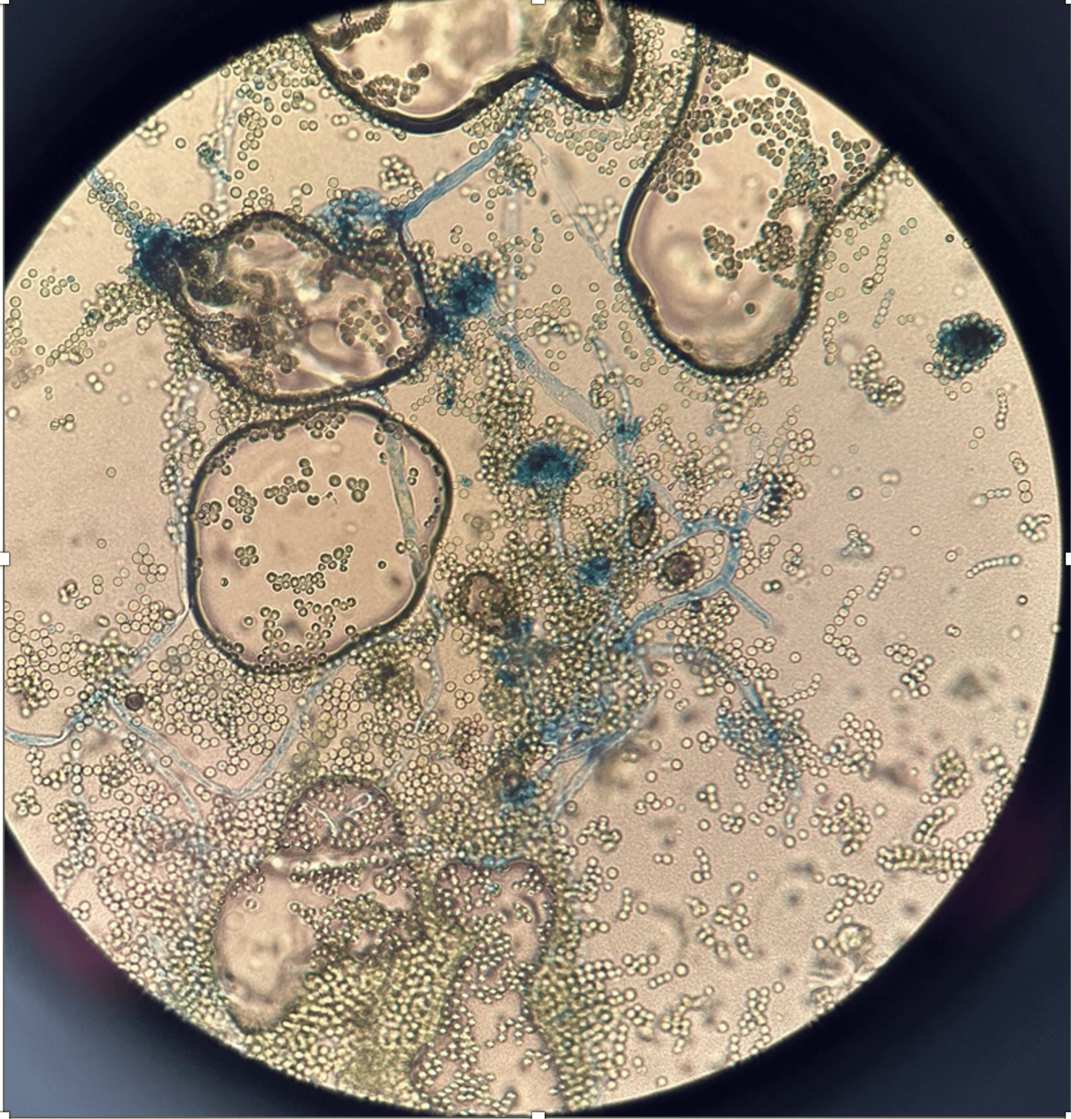
Aspergillus fumigatus microscopic features: uniseriate aspergilli, columnar conidial heads, and flask-shaped vesicles

Mycological analysis of samples revealed filamentous structures and culture-identified colonies of *Aspergillus fumigatus* (Figures 3-4).

The patient was initiated on intravenous voriconazole at 200 mg per day for 14 days, followed by oral therapy for one additional month. The clinical course was remarkable, with clearance of the purulent urine and improvement of laboratory parameters, leading to normalization of renal function and infection markers within approximately 10 days. The patient was followed for six months with no signs of recurrent or persistent fungal colonization.

## Discussion

Urinary tract aspergillosis is a rare condition, primarily observed in patients undergoing kidney transplantation or those with significant immunosuppression [3-5]. Additional contributing factors include bladder catheterization, urinary calculi, vesico-ureteral reflux, and urinary stasis [1]. A recent systematic review of 91 renal and urinary aspergillosis cases extracted from 76 publications spanning 1925-2023 found that the principal risk factors were diabetes mellitus (29.7%), HIV (12.1%), hematological malignancies (11%), and liver cirrhosis (8.8%) [6].

Invasive fungal infections of the urinary tract present significant diagnostic and therapeutic challenges. The infection can occur through three primary pathways: ascending spread, often associated with indwelling bladder catheters; direct inoculation following trauma or surgical procedures; and hematogenous dissemination, which is particularly prevalent in immunosuppressed patients [7]. Our patient had well-controlled diabetes mellitus; however, several other predisposing factors were present, including prior antibiotic therapy, urinary calculi, lithotripsy, and stent placement. These factors likely contributed to fungal colonization by creating a favorable environment for infection.

Our patient had no prior history of urinary tract infection before undergoing instrumentation and ureteral stents placement. While ascending infection remains a possible route, it is more commonly observed in patients with nephrostomy tubes, permanent indwelling devices, or ureteral stents [8].

A case of unilateral renal aspergillosis was reported in a patient with type 2 diabetes following lithotripsy. The authors hypothesized that *Aspergillus *was introduced into the urinary tract during the procedure, likely due to insufficient sterilization of the surgical equipment [9]. Moreover, other cases of renal aspergillosis secondary to renal instrumentation were also found in immunocompetent patients [10]. In our case, there was no direct evidence for the hypothesis regarding contaminated surgical equipment.

Clinically, this condition often presents as a urinary tract infection most often resulting from ascending dissemination. It is nonspecific and may sometimes be associated with urinary tract obstruction or renal failure. Clinical manifestations also vary depending on whether the underlying pathology involves an *Aspergillus *abscess or the formation of a fungus ball. The latter typically results from the aggregation of mucosal debris and foreign or lithiasic material. When a fungal ball obstructs the ureters, patients may present with acute renal colic symptoms. Early diagnosis remains challenging, and direct microscopy or urine culture of fungal aggregates is of major contribution [2,8]. Symptoms depend on the affected region of the urinary tract, manifesting as pyelonephritis, cystitis, or isolated prostatitis [11]. A recent systematic review found that the common symptoms encompass flank pain (36.3%), fever (33%), and lower urinary tract symptoms (20.9%) [6]. Our patient’s clinical presentation was unique due to the pre-existing material in the urinary tract. However, the twice ureteral stents obstruction and the persistent biological inflammatory markers were the major triggers for further explorations. The diagnosis was finally confirmed by pathological examination.

Imaging is essential in the diagnosis of urinary aspergillosis by revealing subtle structural abnormalities that may be missed on clinical examination alone. Renal ultrasound can identify features such as hydronephrosis, focal parenchymal changes, or echogenic patterns suggestive of fungal balls, serving as an accessible and noninvasive initial screening tool. Computed tomography (CT) further enhances diagnostic accuracy by delineating the extent of renal involvement, detecting urinary tract obstructions, and characterizing perinephric inflammatory changes, although its use may be limited in patients with renal insufficiency due to the potential nephrotoxicity of contrast agents. Magnetic resonance imaging (MRI) offers excellent soft tissue resolution without the risk of contrast-induced nephrotoxicity, making it a valuable alternative in patients with compromised renal function. These imaging modalities not only facilitate early and accurate diagnosis but also guide therapeutic decision-making and might help monitor the response to antifungal treatment [12]. Only ultrasound was performed in our case showing mild pyelocaliceal cavities dilation without any evident obstruction. The bilateral renal involvement was responsible for the acute renal failure (ARF), and the cause was twofold: bilateral ureteral obstruction due to the purulent jelly-like texture of urine and probable acute fungal double-sided pyelonephritis.

Ureteroscopy enables direct visualization of the ureteral lumen, revealing fungal masses and subtle mucosal abnormalities that may be overlooked in non-invasive imaging studies. It allows targeted tissue biopsy and collection of specimens, providing definitive histopathological and microbiological diagnosis of urinary fungal infections. Endoscopic intervention facilitates precise removal or fragmentation of obstructing fungal balls, thereby relieving ureteral obstruction and restoring urinary flow. This minimally invasive approach reduces the need for more extensive open surgical procedures and minimizes patient morbidity. Overall, ureteroscopy is indispensable for both accurate diagnosis and effective management of complex urinary tract fungal infections [13].

The mycological examination enables the identification of fungal mycelial aggregates and the determination of the responsible pathogen, along with conducting antifungal susceptibility testing. *Candida albicans* is the most frequently reported pathogen [13]. Some cases of fungal balls caused by *Candida tropicalis *have also been reported [14]. Lastly, *A. fumigatus* is a relatively rare species to be implicated in such infections [15-17].

The management of urinary tract aspergillosis presents a significant therapeutic challenge in clinical practice. Prognosis is generally favorable following endoscopic and antifungal treatment. According to the current guidelines from the Infectious Diseases Society of America, a multidisciplinary approach involving both medical and urological interventions is recommended for renal aspergillosis. Voriconazole is specifically advised for treating parenchymal involvement [3]. First-line antifungal therapy primarily involves azole derivatives (fluconazole, itraconazole, voriconazole) due to their excellent tolerability and bioavailability, tailored to susceptibility testing. Amphotericin B is considered a second-line therapy [18].

## Conclusions

Urinary aspergillosis is a rare fungal infection that poses significant diagnostic and therapeutic challenges due to its nonspecific presentation and insidious onset. The lack of routine fungal screening in urological infections often leads to delayed recognition, increasing the risk of complications such as renal impairment and systemic dissemination. Early clinical suspicion, combined with targeted diagnostic approaches, is essential for timely identification and appropriate antifungal management. Effective treatment requires a multidisciplinary strategy, incorporating risk factor assessment, antifungal therapy, and close follow-up to prevent recurrence. Increased awareness and a systematic diagnostic approach are crucial in improving outcomes for affected patients.
